# Supplementary figures and images for: Rescue of behavioral and electrophysiological phenotypes in a Pitt-Hopkins syndrome mouse model by genetic restoration of Tcf4 expression
Source: eLife. 2022 May 10;11:e72290. doi: 10.7554/eLife.72290 (PMC9090324; doi:10.7554/eLife.72290)

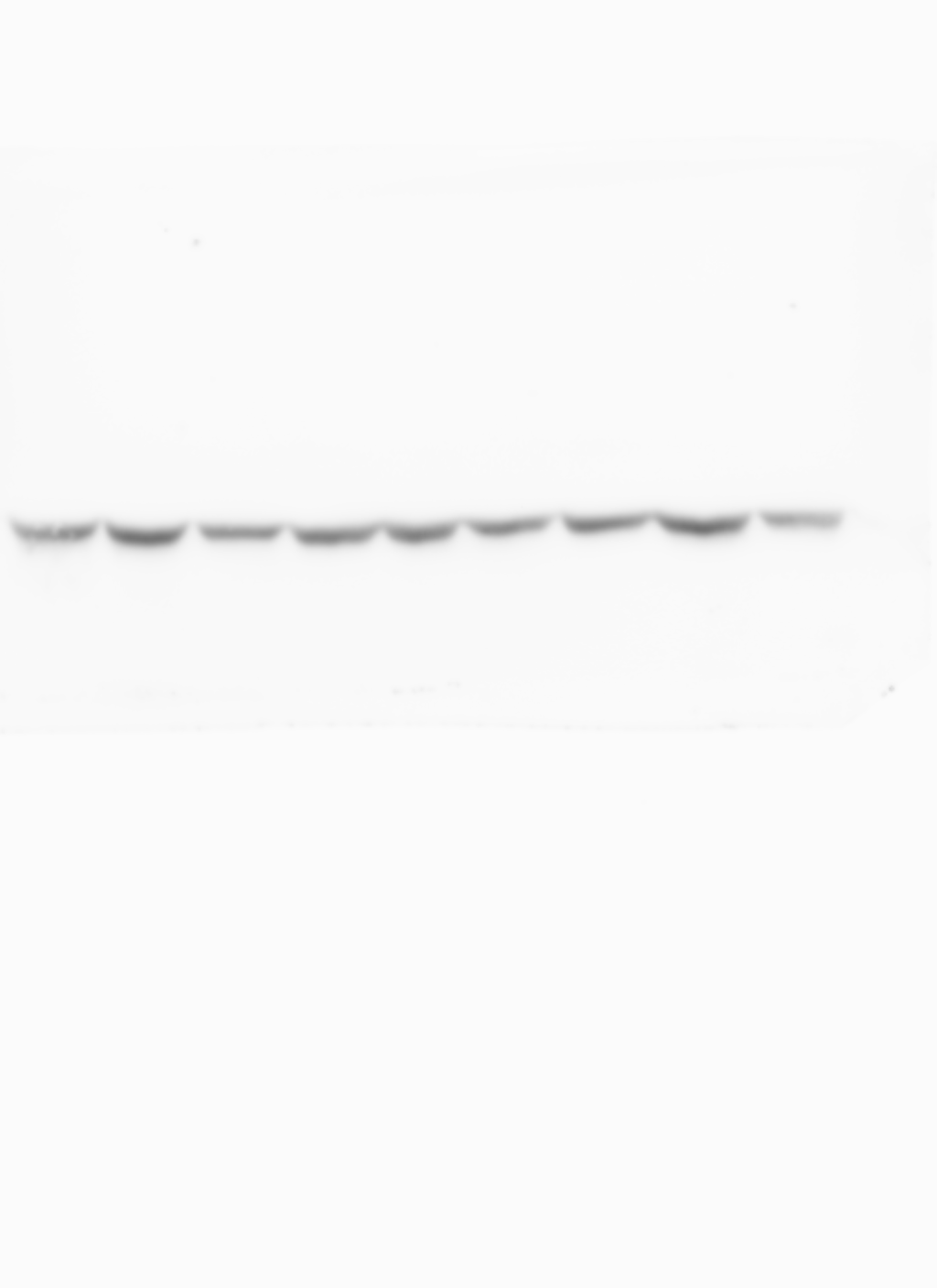

Supplement: Figure 1—source data 1. [file elife-72290-fig1-data1.zip › Figure 1 - source data 1/Beta-tubulin.tif]

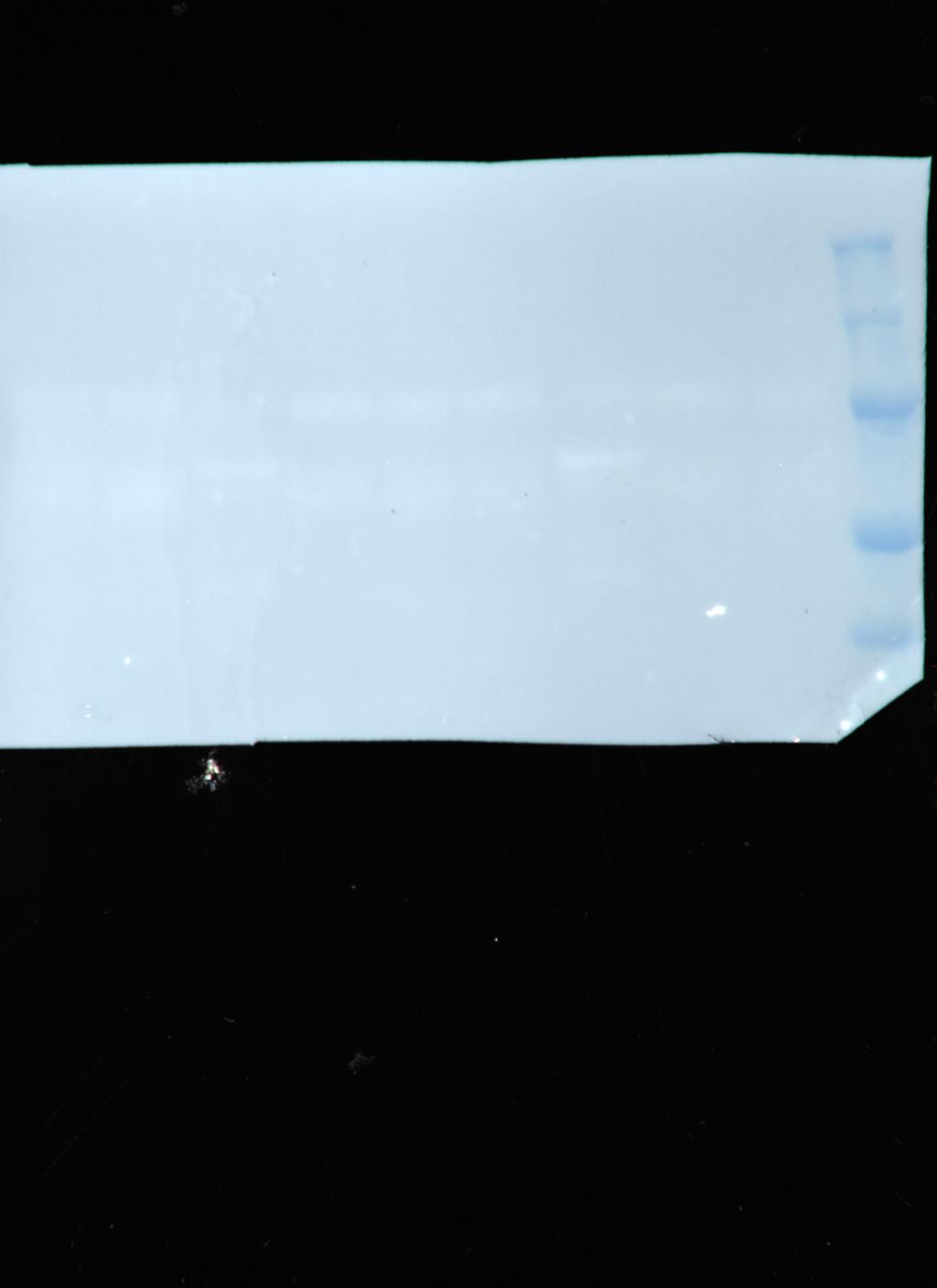

Supplement: Figure 1—source data 1. [file elife-72290-fig1-data1.zip › Figure 1 - source data 1/ladder marker.jpg]

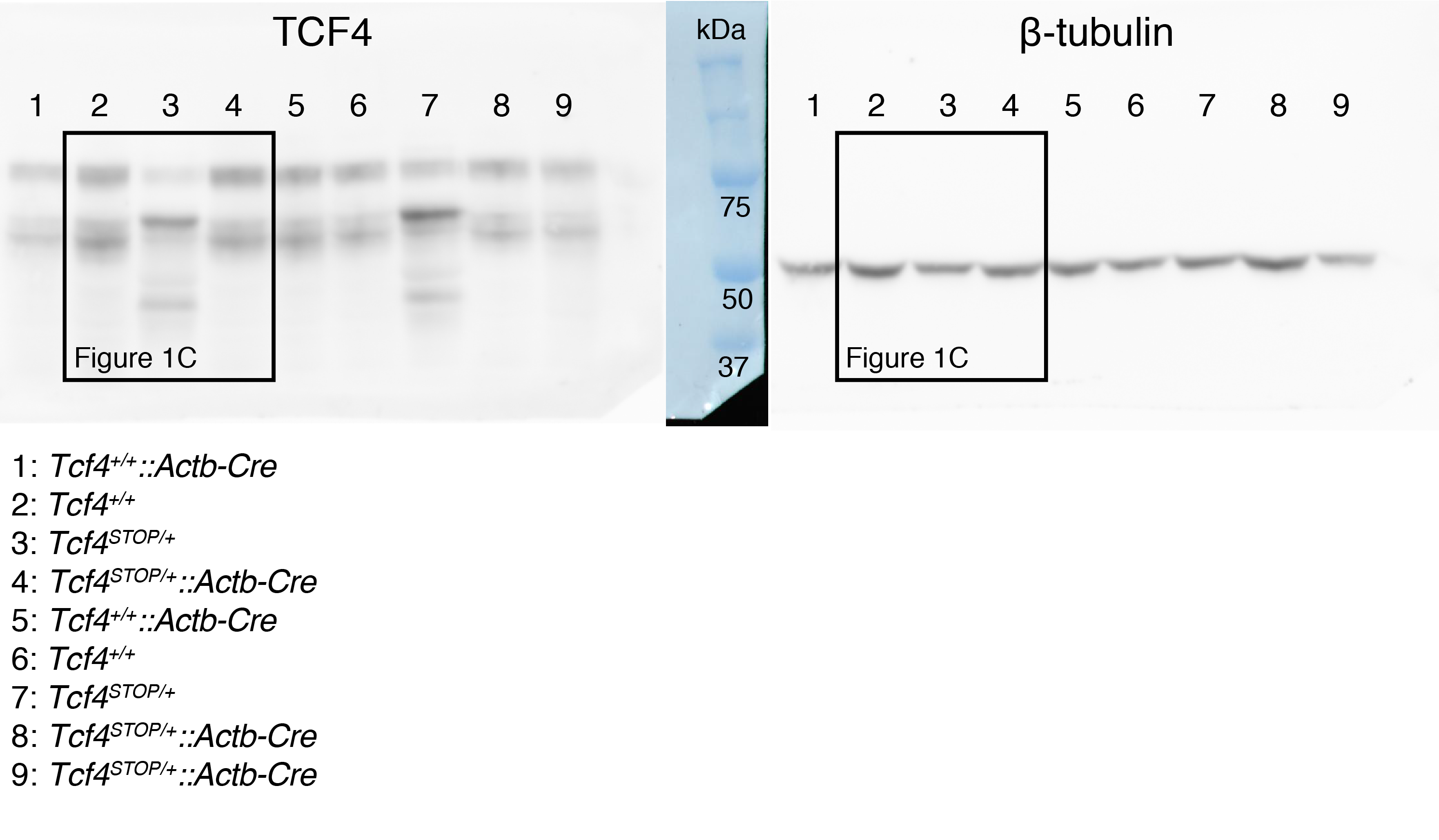

Supplement: Figure 1—source data 1. [file elife-72290-fig1-data1.zip › Figure 1 - source data 1/Figure 1-Source Data 1.tif]

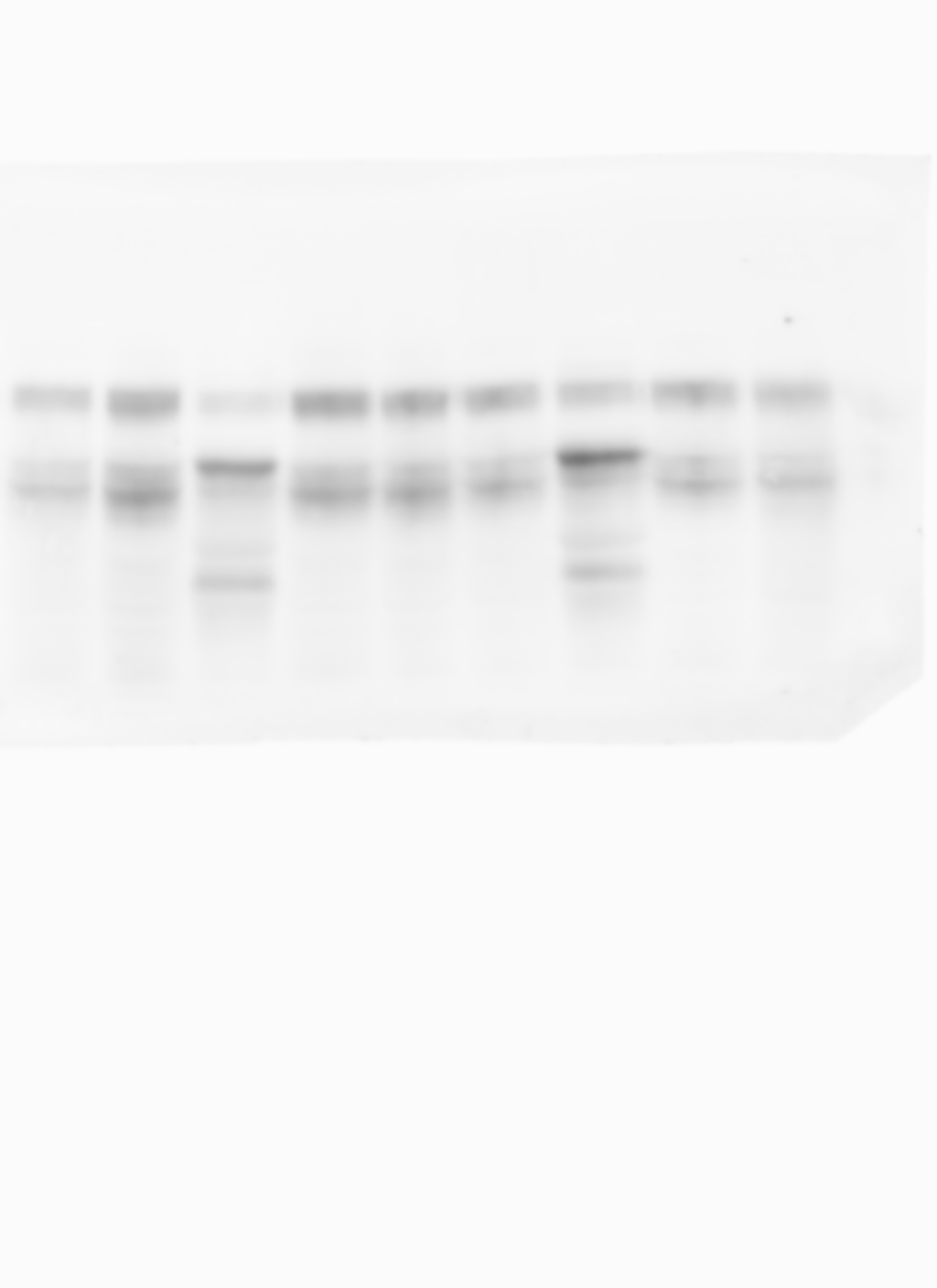

Supplement: Figure 1—source data 1. [file elife-72290-fig1-data1.zip › Figure 1 - source data 1/TCF4.tif]
